# A Multi-Disciplinary Approach for the Management of Prosthetic Joint Infections: An Australian Perspective

**DOI:** 10.5704/MOJ.2207.005

**Published:** 2022-07

**Authors:** JD Sires, K Pham, S Daniel, M Inglis, CJ Wilson

**Affiliations:** 1 College of Medicine and Public Health, Flinders University, Adelaide, Australia; 2 Department of Orthopaedic Surgery, Flinders Medical Centre, Adelaide, Australia; 3 Department of Microbiology and Infectious Diseases, Flinders Medical Centre, Adelaide, Australia

**Keywords:** prosthetic joint infection, revision surgery, multidisciplinary team, total knee arthroplasty, total hip arthroplasty

## Abstract

**Introduction:**

Prosthetic joint infections (PJI) are a major complication of hip and knee arthroplasty, imposing significant morbidity and mortality. Orthopaedic oncology units have utilised a multi-disciplinary team (MDT) approach for some time. PJI is not only an equally life-threatening condition, it also requires input from multiple healthcare personnel and treatment can vary significantly between individuals given the diversity in microbiological, surgical and host factors. Our arthroplasty service established an MDT meeting to manage this complex patient group. This study describes the philosophy and implementation of an MDT approach to the management of PJIs at a tertiary hospital in Australia.

**Materials and methods:**

A retrospective review of all patients that presented to the MDT PJI meeting from October 2017 to April 2020 was performed. Patient characteristics, microbiological profile and management were reviewed.

**Results:**

One hundred and one patients were reviewed over 2.5 years with a mean age of 69.2 years (SD 11.9). Patients presenting predominantly had a primary TKR (32%) or primary THR (22%). Results of Microbiology cultures varied, with 42% Gram-positive organisms, 13% Gram-negative organisms, 2% fungus and 1% yeast origin. Management mainly consisted of two-stage revision (28%), debridement-antibiotics-and-implant retention (22%) and antibiotic suppression (14%). A total of 91.5% of patients who underwent surgical management were considered cured at one year.

**Conclusion:**

PJIs are complex and require coordinated care by a number of healthcare personnel. The MDT process has allowed collaboration between Orthopaedic, Infectious Disease and Microbiology departments and aims to improve the quality of care provided to patients, potentially reducing morbidity and mortality of patients with PJI.

## Introduction

Prosthetic joint infections (PJI) are a major complication of hip and knee arthroplasty, and although rare (1% - 2%) become an increasing problem with the cumulative amount of hip and knee replacements that are performed each year^1,2^. Morbidity and mortality for PJI is high, with rates of 11% at 1 year and 26% at 5 years for individuals that undergo at least one surgical procedure for a PJI^3^. Orthopaedic oncology units have been utilising a multi-disciplinary team (MDT) approach for some time and have shown to increase patient survival rates^4^. PJIs are not only an equally life-threatening condition compared to some cancers^3^, they can also be just as complex due to the wide variation in host factors, microbiological profile and surgical complexity, and therefore require input from a number of specialists. Additionally, there is currently no accepted international guideline for the treatment of PJIs, with various groups worldwide suggesting different management approaches according to certain criteria, however, these can be difficult to apply to certain individuals given the diverse presentation. The idea of an MDT approach to treating this complex cohort of patients has been suggested, with some overseas groups publishing their early results^5-8^.

The aim of this study was to discuss the development and philosophy of our MDT team’s approach to the management of PJI in an Australian tertiary referral hospital. Additionally, we aim to present the concept of a prospective database for PJIs, which focuses on patient demographics, microbiological profile and management of patients to date.

An MDT PJI meeting was developed at our tertiary hospital at Flinders Medical Centre in 2017, with the aim of providing optimal management to this complex patient group. At the time of its establishment, there was no dedicated PJI MDT meeting in South Australia, and with growing awareness among both Orthopaedic and Infectious disease doctors locally regarding the challenges of complex PJI the MDT meeting was established. Additionally, a prospective database was established for patients that were reviewed in the PJI MDT meeting, allowing demographics, microbiological profile, management and outcomes to be reviewed on an ongoing basis. Our PJI MDT meeting occurs monthly and discusses both newly referred cases and the progress of previously discussed patients. Inclusion criteria for referral include a suspected PJI, infected metal work after trauma surgery and septic arthritis of the hip and knee. Both public and private patients’ cases are discussed collectively. Staff involved in our MDT include; orthopaedic surgeons who specialise in revision arthroplasty, infectious disease physicians with significant musculoskeletal infection experience, senior registrars, microbiology registrar, junior doctors and a nurse consultant. Plastic surgeons, nutritionists and physiotherapists are consulted on a case-to-case basis. Additionally, as many of the patients with PJIs have multiple comorbidities, there is the option to refer patients to other specialist units for pre-operative optimisation or postoperative care if needed.

Overall, the philosophy of the PJI MDT meeting is to provide a coordinated service involving all stakeholders, creating a service that provides the best results to patients. Before the meeting, the senior registrar Orthopaedic surgeon prepares a handout of patients that will be discussed. This includes information on patient demographics, past medical history, index arthroplasty case, lab results, aspiration/arthroscopic biopsy results, previous antibiotic/surgical management and planned follow-up and is emailed to all the members of the MDT. Additionally, the document often includes any specific management questions that need to be discussed at the MDT. The senior fellow or junior doctor concerned presents the case followed by an expert opinion from the panel of revision arthroplasty surgeons. The lead Infectious disease and Microbiology registrar discusses the available Microbiology results, the need for further investigation if any culminating in a provisional antimicrobial. This often leads to a discussion on various management options, listing the pros and cons of each, and arriving at the best management plan for the individual. This is then conveyed to the patient and bookings made, the process facilitated by a nurse consultant who attends each meeting, who is often the first contact for patients, ensuring a smooth transition from discussion to management.

## Materials and Methods

This was a single centre, retrospective review of all cases discussed in the MDT PJI meeting at Flinders Medical Centre between its commencement in October 2017 and April 2020. An MDT proforma was used to collect information on patient demographics, past medical history, American Society of Anaesthesiologist (ASA) grade, body mass index (BMI), index arthroplasty case, lab results, aspiration/arthroscopic biopsy results, previous antibiotic/surgical management, and planned follow-up. Data were entered into an excel file and analysed. Data were analysed using SPSS version 19 [IBM, NY]. Primary outcomes included patient demographics, joint/implant involved, microbiological profile, as well as completed and/or planned management. Individuals who underwent surgical management of a prosthetic joint infection had their Delphi criteria determined as per Diaz-Ledezma (2013) *et al*^9^; (1) infection eradication, characterised by a healed wound without fistula, drainage, or pain, and no infection recurrence caused by the same organism strain, (2) no subsequent surgical intervention for infection after reimplantation surgery, and (3) no occurrence of PJI-related mortality (by causes such as sepsis, necrotising fasciitis). Individuals meeting all Delphi criteria at one year postoperatively were considered cured. Proportions, means and standard deviations were calculated. An ethics review was not sought because the study met the criteria for exemption from such review according to an institutional policy.

## Results

A total of 162 cases were reviewed in the PJI MDT meeting over a 2.5-year period, with a mean age of 69.2 (SD 11.9) years and 55% being female. This involved 101 individual patients, who were discussed between 1 and 7 times. The patient past medical history included diabetes (24%), inflammatory arthropathy (19%), chronic obstructive pulmonary disease (14%), chronic kidney disease (11%), and heart failure (11%). BMI was available for 80 patients, with 59% considered obese, BMI >30. The average ASA grade was 2.68, with 65% being >3. Patients presenting predominantly had a primary total knee replacement (TKR) (32%), followed by a primary total hip replacement (THR) (22%) at presentation to the PJI MDT meeting. In total, 18% of individuals had previously undergone revision arthroplasty as shown in Table I.

**Table I:** Breakdown of the presenting joint and/or implant at presentation to PJI MTD meeting

Presenting Joint and/or Implant	n (%)
Native Hip	6 (5.9%)
Native Knee	9 (8.9%)
Primary Hip	22 (21.8%)
Primary Knee	32 (31.7%)
Revision Hip	12 (11.9%)
Revision Knee	6 (5.9%)
Primary Elbow	2 (2.0%)
Trauma	12 (11.9%)

Microbiological cultures were completed in 91 patients (90%), with the most recent positive results being highlighted in Table II. Cultured organisms varied significantly among patients, with 42% having Gram positive, 13% Gram negative, 2% fungal and 1% yeast in origin. In total, 7 (8%) had more than one organism isolated, 12 (13.2%) of cases were deemed a suspected contaminant, and 28 (30.8%) cases produced nil growth after culture completion.

**Table II:** Microbiological profile of most recent culture of patients presenting to PJI MTD meeting

Total Cultures (n = 91)	n (%)
Gram-positive	38 (41.8%)
* MS Staphylococcus aureus*	9 (9.9%)
* Staphylococcus epidermis*	7 (7.7%)
* MRSA*	6 (6.6%)
* Other*	20 (22.0%)
Gram-negative	12 (13.2%)
* Pseudomonas spp.*	7 (7.7%)
* Escherichia coli*	2 (2.2%)
* Other*	3 (3.3%)
Fungus	2 (2.2%)
* Aspergillus fumigatus*	1 (1.1%)
* Cryptococcus neoformans*	1 (1.1%)
Yeast	1 (1.1%)
* Candida albicans*	1 (1.1%)
Multiple Organisms	7 (7.7%)
Suspected Contaminant	12 (13.2%)
Nil Growth	28 (30.8%)
Nil cultures available	10 (11.0%)

Completed or planned management of patients seen in the PJI MDT meeting are highlighted in Table III. Management predominantly consisted of two-stage revision (28%) and debridement-antibiotic-implant retention (DAIR) (22%), with some patients receiving only antibiotic suppression (14%). A small group of patients underwent single-stage revision arthroplasty (4%), with many patients having ongoing clinical review (24%).

**Table III:** Planned/Completed Management of patients presenting to PJI MTD meeting

Planned/Completed Management	n (%)
Debridement-Antibiotic-Implant Retention	22 (21.8%)
One stage revision arthroplasty	4 (4.0%)
Two stage revision arthroplasty	28 (27.8%)
Primary joint arthroplasty	9 (8.9%)
Ongoing Clinical Review	24 (23.8%)
Antibiotic suppression	14 (13.9%)

The overall mortality rate was high (13%), with 5 out of the 13 were related to a PJI, with 3 of these patients declining further surgery during hospitalisation and subsequently receiving palliative therapy. The proportion of patients who underwent surgical management (n = 69) and were considered cured at one year as per Delphi Criteria (excluding death not related to PJI) was 92%, this was 90% for those with an acute infection and 95% for those with a chronic infection.

## Discussion

This preliminary review of the PJI MDT approach reveals a broad spectrum of patients with joint/implant infection, varying microbiological profiles and management options. Given the aforementioned diversity, and complexity of such a condition, a multi-disciplinary approach is warranted. MDT meetings for the management of orthopaedic related tumours have been the standard of care for the past two decades, and as suggested by previous studies should also be the standard of care for prosthetic joint infections^7^.

Literature on other centres that have established and published on their experiences of an MDT for management of PJIs is scarce, however, practice is thought to be growing. Akgün (2019) *et al*^5^ discussed an MDT approach to the management of hip PJIs managed using two-stage revision in a series of 93 cases, and utilised the expertise of orthopaedic surgeons, infectious disease specialists as well as internal medicine specialists. They also discussed how the importance of medical optimisation of patients as a higher Charlson Comorbidity Index was associated with increased risk of reinfection and mortality. Additionally, a study by Ibrahim (2014) *et al*^6^ published results on their management utilising an MDT in the management of patients presenting with PJI of the hip and found good eradication rates at 5 years at 96%. A study by Ntalos (2019) *et al*^7^ looked at outcomes pre and post establishment of an MDT team approach and found patients discussed in the MDT conference had a shorter length of stay, fewer surgeries, and a smaller number of antibiotics used. They also discussed the workflow of their MDT, which differed from ours in the following ways; their meeting was weekly, whereas ours was monthly, they also discussed osteomyelitis, soft tissue infections and osteosynthesis, whereas we also discussed some native septic arthritis cases as well as trauma cases. Lastly, one group compared patients undergoing 2 stage exchange for a PJI of the hip or knee and found those treated after the interdisciplinary team progressed to their second stage quicker and had reduced recurrence of infection^10^. This group also discussed their standardised follow-up of these patients, which included outpatient Infectious disease department review at 6, 12, 26, and 52 weeks.

A PJI MDT meeting offers a number of benefits to staff and patients. Coordinated care between Orthopaedic surgeons and infectious disease specialists allows patient-specific management in a timely manner, with complex decision-making being shared and agreed upon. Additionally, the inclusion of a nurse consultant as a central coordinator can ensure involved staff and patients are aware of upcoming appointments, surgery dates and changes to treatment that may occur. Other benefits include the creation of expertise as high case numbers are discussed, as well as being a great learning environment for trainee surgeons and physicians. Additionally, we experienced multiple challenges when establishing our PJI MDT meeting. This included minimal published research on MDT meeting use in PJI and optimal management of PJIs, mobilising different groups of medical experts to meet together and lack of a local national model to follow.

Currently, plans are underway to increase the effectiveness of our PJI MDT meeting. This includes consideration of adding a pathologist, radiologist, nutritionist and vascular surgeon to the group on a more regular basis. The development of a local one vs two-stage protocol is underway and a six-monthly evaluation of patient outcomes through our local PJI database is planned. There are plans for a larger audit to include all patients who have had MDT input for the management of PJI in the Southern Adelaide Local Health Network area specifically looking for clinical outcomes. Additionally, the literature suggests a number of modifiable risk factors exist which are associated with increased rates of PJI such as diabetes severity, smoking, obesity and malnutrition^11,12^. A more targeted pre-operative medical optimisation of these factors including specific general practitioner follow-up or referral to respective medical specialities before definitive surgery may help in achieving better outcomes.

This is the first study discussing the MDT approach to the management of PJI from an Australian/New Zealand perspective. We feel this approach has assisted in the coordination, streamlining and standardisation of our processes in the management of PJIs, and recommend such an approach in tertiary hospitals. Additionally, we have described a way in which local centres can set up a process and monitor their data, which can be used in a number of ways such as identifying local patterns in infections and also developing local guidelines. Lastly, we have discussed the benefits, challenges and future improvements of a PJI MDT meeting, allowing those who are thinking of establishing an MDT meeting area to improve upon allowing a smoother transition.

Our study included several limitations. Firstly, this was a preliminary analysis of the PJI MDT approach at our institution and did not include the analysis of clinical outcomes. Secondly, we have included a mixture of acute and chronic infections, as well as a broad range of presenting joints and implants, and given the relatively small sample size of our cohort, we are unable to further analyse each subgroup.

## Conclusion

Prosthetic joint infections are complex and require coordinated care by a number of healthcare personnel. The MDT process has allowed collaboration between Orthopaedic and Infectious Disease, Microbiology, allied health and nursing departments and aims to improve the quality of care provided to patients, therefore potentially reducing the morbidity and mortality of patients with prosthetic joint infections. The construction of a local prospective database allows monitoring of both short and long-term outcomes of management.

## References

[1] Lamagni T (2014). Epidemiology and burden of prosthetic joint infections.. J Antimicrob Chemother..

[2] Lenguerrand E, Whitehouse MR, Beswick AD, Toms AD, Porter ML, Blom AW (2017). Description of the rates, trends and surgical burden associated with revision for prosthetic joint infection following primary and revision knee replacements in England and Wales: an analysis of the National Joint Registry for England, Wales, Northern Ireland and the Isle of Man.. BMJ Open..

[3] Zmistowski B, Karam JA, Durinka JB, Casper DS, Parvizi J (2013). Periprosthetic joint infection increases the risk of one-year mortality.. J Bone Joint Surg Am..

[4] Blay JY, Soibinet P, Penel N, Bompas E, Duffaud F, Stoeckle E (2017). Improved survival using specialized multidisciplinary board in sarcoma patients.. Ann Oncol..

[5] Akgün D, Müller M, Perka C, Winkler T (2019). High cure rate of periprosthetic hip joint infection with multidisciplinary team approach using standardized two-stage exchange. J Orthop Surg Res..

[6] Ibrahim MS, Raja S, Khan MA, Haddad FS (2014). A multidisciplinary team approach to two-stage revision for the infected hip replacement: a minimum five-year follow-up study.. Bone Joint J..

[7] Ntalos D, Berger-Groch J, Rohde H, Grossterlinden LG, Both A, Luebke A (2019). Implementation of a multidisciplinary infections conference affects the treatment plan in prosthetic joint infections of the hip: a retrospective study.. Arch Orthop Trauma Surg..

[8] Sukeik M, Haddad FS (2019). Periprosthetic joint infections after total hip replacement: an algorithmic approach.. SICOT J..

[9] Diaz-Ledezma C, Higuera CA, Parvizi J (2013). Success after treatment of periprosthetic joint infection: a Delphi-based international multidisciplinary consensus. Clin Orthop Relat Res..

[10] Karczewski D, Winkler T, Renz N, Trampuz A, Lieb E, Perka C (2019). A standardized interdisciplinary algorithm for the treatment of prosthetic joint infections.. Bone Joint J.

[11] Alamanda VK, Springer BD (2019). The prevention of infection: 12 modifiable risk factors.. Bone Joint J..

[12] Wilson CJ, Georgiou KR, Oburu E, Theodoulou A, Deakin AH, Krishnan J (2018). Surgical site infection in overweight and obese Total Knee Arthroplasty patients.. J Orthop..

